# Dihydroartemisinin: A Potential Natural Anticancer Drug

**DOI:** 10.7150/ijbs.50364

**Published:** 2021-01-16

**Authors:** Xiaoshuo Dai, Xiaoyan Zhang, Wei Chen, Yihuan Chen, Qiushuang Zhang, Saijun Mo, Jing Lu

**Affiliations:** 1 Department of Pathophysiology, School of Basic Medical Sciences, Zhengzhou University, Zhengzhou, Henan Province 450001, PR China; 2 Collaborative Innovation Center of Henan Province for Cancer Chemoprevention, Zhengzhou University, Zhengzhou, Henan Province 450001, PR China; 3 State Key Laboratory of Esophageal Cancer Prevention & Treatment, Zhengzhou University, Zhengzhou, Henan Province 450052, PR China

**Keywords:** Dihydroartemisinin, Cancer, Proliferation, Apoptosis, Combination medication, Clinical trail

## Abstract

Dihydroartemisinin (DHA) is an active metabolite of artemisinin and its derivatives (ARTs), and it is an effective clinical drug widely used to treat malaria. Recently, the anticancer activity of DHA has attracted increasing attention. Nevertheless, there is no systematic summary on the anticancer effects of DHA. Notably, studies have shown that DHA exerts anticancer effects through various molecular mechanisms, such as inhibiting proliferation, inducing apoptosis, inhibiting tumor metastasis and angiogenesis, promoting immune function, inducing autophagy and endoplasmic reticulum (ER) stress. In this review, we comprehensively summarized the latest progress regarding the anticancer activities of DHA in cancer. Importantly, the underlying anticancer molecular mechanisms and pharmacological effects of DHA *in vitro* and* in vivo* are the focus of our attention. Interestingly, new methods to improve the solubility and bioavailability of DHA are discussed, which greatly enhance its anticancer efficacy. Remarkably, DHA has synergistic anti-tumor effects with a variety of clinical drugs, and preclinical and clinical studies provide stronger evidence of its anticancer potential. Moreover, this article also gives suggestions for further research on the anticancer effects of DHA. Thus, we hope to provide a strong theoretical support for DHA as an anticancer drug.

## Introduction

Artemisinin, which is derived from the annual Compositae family member *Artemisia annua L.*, has been used as a traditional Chinese medicine for more than 2,000 years, and dihydroartemisinin (DHA) is the first generation derivative of this compound. Artemisinin and its derivatives (ARTs) have cured more than one million malaria patients 1, and Tu Youyou, who made outstanding contributions to this field, has won several awards, including the Lasker-DeBakey Clinical Research Award in 2011 and the Nobel Prize in Medicine or Physiology in 2015 2. In 1973, Tu Youyou's research group synthesized DHA by reducing artemisinin with sodium borohydride. The hydroxyl group in the structure of DHA not only improved the antimalarial activity but also served as an entry point for the synthesis of a series of ARTs. The antimalarial drugs developed based on artemisinin include artesunate, artemether and arteether 3. The result of drug metabolism study shows that the primary active metabolites of artesunate, artemether and arteether is DHA, which indicates that these drugs primarily play a role *in vivo* through the production of DHA (the original drug) 4.

**Figure 1:**
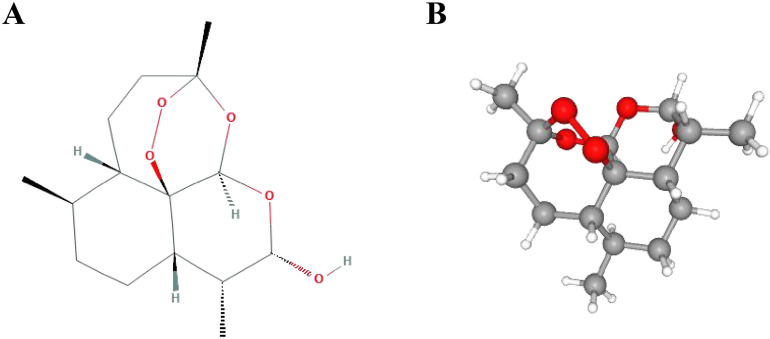
Chemical structures of dihydroartemisinin. (A) 2D structure. (B) 3D conformation.

DHA, with a molecular formula of C_15_H_24_O_5_ and a molecular weight of 284.35 (Figure 1), has been demonstrated to be an effective and fast-acting antimalarial drug with low toxicity. Compared with artemisinin, DHA has better water solubility and stronger antimalarial activity, with antimalarial efficacy that is more than 10 times that of artemisinin. In addition, the recurrence rate of disease after treatment with DHA is as low as 1.95%. After the huge success of DHA in treating malaria in the early 1990s, Tu Youyou's group began to explore new indications for DHA, and made great progress in the research of systemic lupus erythematosus 5, which suggests that DHA may have other potential indications besides its anti-malaria effects.

Cancer is the largest threat to global human health 6. Currently, radiation therapy, chemotherapy and combination therapy are the main methods in tumor treatment 7. However, chemotherapy and radiotherapy have notable toxicity and side effects on healthy tissues, which adversely affect the effect of treatment to some extent 8. Therefore, there is an urgent need to develop new and effective anti-tumor drugs. It is worth noting that DHA has received increasing attention for its anticancer function due to its low toxicity and known safety. This review provides a comprehensive overview of anticancer roles of DHA, highlights the molecular mechanisms and pharmacological effects* in vitro* and* in vivo* as well as combination medication, discusses the improvement designs and clinical trials, and hopes to provide reference and inspiration for further exploration of DHA.

## The anticancer effects of DHA

**Figure 2:**
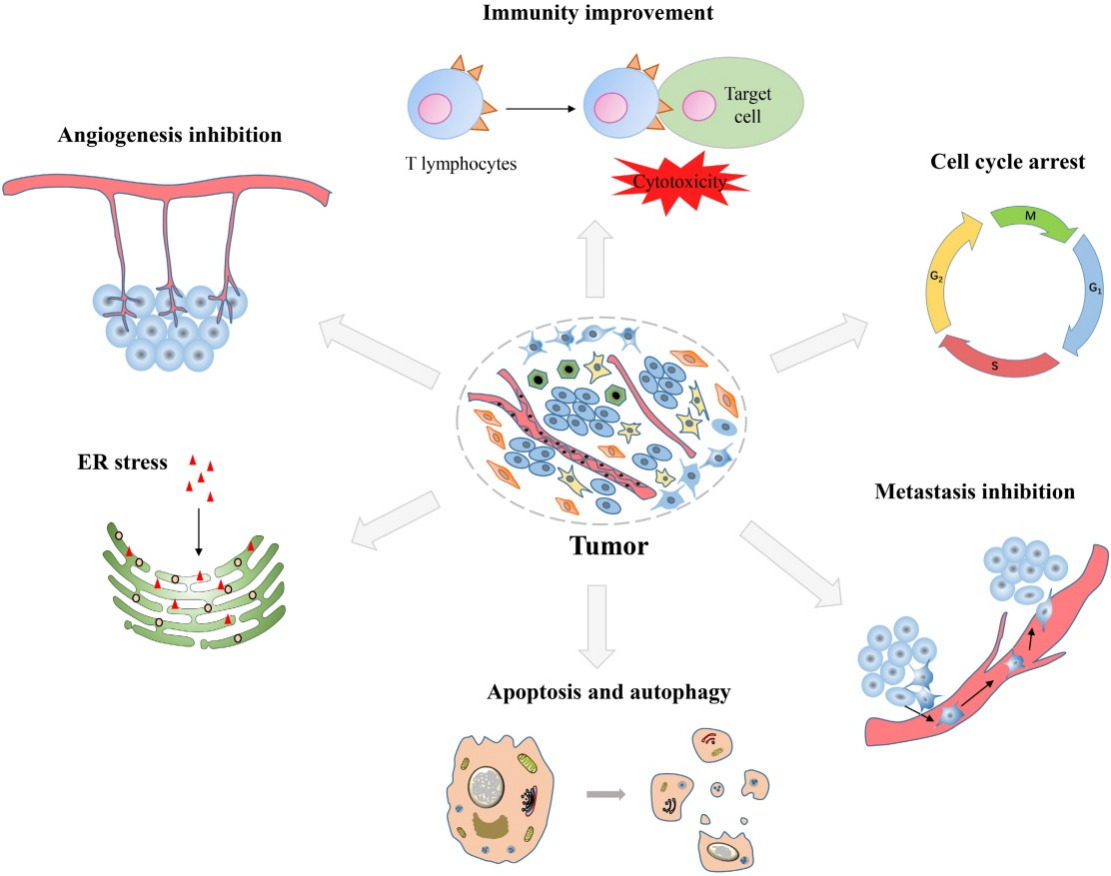
Synopsis of the mechanisms of dihydroartemisinin action against tumor cells.

DHA has displayed anticancer effects on many types of tumors, such as lung cancer, breast cancer, prostate cancer, ovarian cancer and digestive system tumors. In general, DHA has been proven to have many anticancer effects, including inhibiting proliferation, inducing apoptosis, inhibiting tumor metastasis and angiogenesis, promoting immune function, inducing autophagy and endoplasmic reticulum (ER) stress (Figure 2). Herein, we systematically summarized the anticancer effects of DHA, with emphasis on the pharmacological effects and molecular mechanisms, so as to make a brief description of the anticancer effects of DHA.

### Inhibition of proliferation

Cell division is necessary for the growth and repair of normal cells, however, uncontrolled cell division is one of the basic characteristics of cancer cells 9. In general, DNA damage induced by drug would greatly affect cell integrity, interrupt the process of cell replication and division, and ultimately lead to cell cycle arrest and cell death. DHA has been shown to have powerful anti-tumor effects by inhibiting cancer cell proliferation *in vitro* and* in vivo*.

Cell cycle is the basic process of cell life, and the arrest of cell cycle can directly inhibit cell growth. Haijun Sun et al. observed that DHA inhibited the growth of gastric cancer cells (MGC803, BGC823 and SGC7901) with downregulating the expression of some proliferation markers (PCNA, Cyclin E and Cyclin D1), besides, DHA was also shown to be capable of inducing cell cycle arrest in the G1 phase 10. Furthermore, DHA upregulated the expression of Bax and led to caspase-9 activation, and downregulated the expression of Bcl-2, Bcl-x_L_, Cyclin E, CDK2 and CDK4, which led to esophageal cancer cell cycle arrest 11. Kui Liao et al. observed that DHA inhibited the growth of A549 cells by inhibiting the protein kinase B (AKT)/glycogen synthase kinase-3β (Gsk3β)/Cyclin D1 signaling pathway and led to cell cycle arrest in the G1 phase 12. Besides, DHA could inhibit the proliferation of hepatocellular carcinoma (HCC) cells by inducing cell cycle arrest in G2/M phase 13.

**Figure 3:**
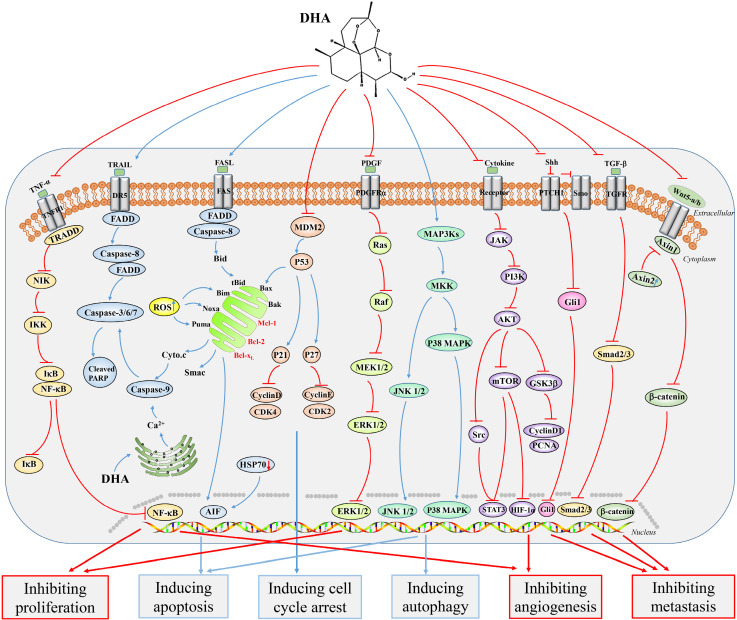
Major signaling pathways involved in the anticancer effects of dihydroartemisinin. Dihydroartemisinin can inhibit the NF-κB, TGF-β, hedgehog, PI3K/AKT/HIF-1κ, JAK2/STAT3, Wnt/β-catenin, AKT/GSK-3β/Cyclin D1, and AKT/mTOR/STAT3 signaling pathways. In addition, dihydroartemisinin can activate the JNK1/2, p38 MAPK, death receptor-mediated and mitochondrion-mediated caspase-dependent apoptotic signaling pathways. Red curves indicate inhibition and blue arrows indicate activation of these processes.

Remarkably, DHA also has strong anti-tumor effects *in vivo*. DHA could inhibit the growth of SGC7901 xenograft model, as well as Eca109 and Ec9706 xenograft tumors 10, 14. Furthermore, treating mice with DHA at a dose of 20 mg/kg could effectively inhibit colon tumor growth compared to those treated with the control regimen 15. Moreover, Peng Han et al. firstly revealed that knocking down the small GTPase Rac1 could strengthen the growth inhibition and cell cycle arrest induced by DHA in HCT116 and RKO colon cancer cell lines. Besides, when DHA was combined with Rac1 siRNA *in vivo*, it exhibited a significantly enhanced anti-tumor effect. In summary, Rac1 siRNA could promote the anticancer effect of DHA towards colon cancer by inhibiting nuclear factor kappa B (NF-κB) activation 16 (Figure 3).

Excessive reactive oxygen species (ROS) will exert a toxic effect on cell and affect the growth of normal cells. Diancheng Wang et al. found that DHA could increase the level of ROS in cells, thereby exerting a cytotoxic effect in cancer cells. When treated with kidney form of glutaminase (GLS1) inhibitor 968, ROS could not be eliminated in HCC cells, which made HCC cells more sensitive to DHA-mediated cytotoxicity. Hence, these results provided a basis for the clinical treatment of liver cancer by targeting glutamine metabolism and binding the ROS generator DHA 17. Interestingly, an investigation by Tao Su et al. revealed DHA could inhibit the growth of *H. pylori* and gastric cancer cells by NF-κB signaling. Besides, DHA suppressed *H. pylori* adhesion to the gastric cancer cells and reduced the *H. pylori*-enhanced ROS production, and may have a therapeutic effect on *H. pylori*-induced gastric cancer 18.

### Induction of apoptosis

Abnormal cell proliferation is related to the disorder of apoptosis regulation, and the reduction or loss of apoptosis ability can lead to the infinite proliferation and metastasis of tumor cells. In general, tumor cells can escape from apoptosis and keep on survival and growth by down-regulating pro-apoptotic factors and up-regulating anti-apoptotic factors 19. Apoptosis pathways include exogenous pathways mediated by death receptors and endogenous pathways mediated by mitochondria.

Current studies have shown that DHA can significantly induce apoptosis of tumor cells *in vitro* and* in vivo*. Haiting Mao et al. studied that DHA effectively inhibited the proliferation of T-47D breast cancer cells by increasing the protein expression of caspase-8, cleaved caspase-9 and Bim, activating Bid and inducing cytochrome c release. These results indicated that the mitochondrial pathway exerted an important effect in the process of DHA-induced breast cancer cell apoptosis, and the imbalance of Bim/Bcl-2 interactions promoted this process 20 (Figure 3). Bim and a small amount of Noxa could activate Bak-mediated intrinsic apoptotic pathways as upstream mediators 21. Furthermore, DHA induced caspase-dependent apoptosis in HCC SK-Hep-1 cells through proteasome-dependent degradation of specificity protein 1 (Sp1) 22. Several signaling pathways were found to be significantly associated with DHA-induced apoptosis. DHA induced the apoptosis of colon cancer cells by targeting janus kinase 2 (JAK2)/signal transducer and activator of transcription 3 (STAT3) signaling 23. Furthermore, NF-κB signaling pathway was also shown to play an important role in the process of DHA-induced apoptosis 24-26. Another study reported that DHA induced apoptosis in BGC823 gastric cancer cells by c-Jun NH_2_-terminal kinases (JNK1/2) and p38 mitogen-activated protein kinase (p38 MAPK) signaling pathways 27. In addition, the activation of Ca^2+^ and p38 was also observed in DHA-induced apoptosis of PC14 lung cancer cells 28 (Figure 3). More importantly, DHA was also shown to be able to induce apoptosis in ovarian cancer cells, and compared with a series of ARTs, DHA had the strongest effect 29.

Some molecular targets of DHA-induced apoptosis of tumor cells were also found. Maria Lucibello et al. found that DHA induced apoptosis by targeting a phosphorylated form of translationally controlled tumor protein (TCTP). Subsequently, the clinical data showed nuclear phosphorylation of TCTP was increased in primary breast cancer tissues compared with that seen in normal tissues, which was accompanied by higher histological grade and increased Ki-67 expression. These results suggested that phosphorylated TCTP might be a potential target of DHA in the treatment of advanced breast cancer 30. Ge Xu et al. revealed that the down-regulation of heat-shock protein 70 (HSP70) might participate in the apoptosis of PC3 prostate cancer cells induced by DHA via proteomics analysis 31. In addition, Jin Kong et al. demonstrated through experiments that DHA could inhibit the expression of HSP70 and induce apoptosis in PC3 cells 32. Furthermore, DHA inhibited the growth of colon tumor by inducing apoptosis and increasing the expression of peroxisome proliferator-activated receptor γ (PPARγ) 33.

Metabolic pathways are also closely related to apoptosis. DHA was shown to inhibit the activity of glucose transporter-1 (GLUT1) and glycolytic pathway by inhibiting phosphatidyl-inositol-3-kinase (PI3K)/AKT pathway and downregulating the expression of hypoxia inducible factor-1α (HIF-1α), thereby inducing LNCaP cell apoptosis 34. Pyruvate kinase M2 (PKM2) is a key regulator of glycolysis, and a study by Shumin Li et al. reported that PKM2 expression was higher in esophageal squamous cell carcinoma than in normal tissues. Interestingly, DHA could inhibit the expression of PKM2 as well as inhibit lactic acid production and glucose uptake, thereby promoting the apoptosis of esophageal cancer cells 35. Furthermore, DHA induced caspase-independent apoptosis-like cell death in Colo205, HCT15 and HCT116 colorectal cancer cells in the absence of oxygen. Notably, normoxic and hypoxic conditions did not make a significant difference in this effect. Thus, the results demonstrated that DHA showed significant cytotoxicity in severe hypoxia and normoxic environments, which provided a new perspective for the treatment of tumor cells in hypoxia 36. The above results indicated that DHA could induce apoptosis of tumor cells by regulating the metabolic process.

### Inhibition of metastasis

Tumor metastasis refers to the process by which cancer cells escape from the primary tumor and break down the extracellular matrix, eventually reaching other sites for further growth 37. Despite the progress that has been made in the treatment of cancer, there have been no substantial reductions in the high mortality rate in the past few years, primarily because of the metastasis of cancer cells 38, 39. In clinical treatment, tumor metastasis will greatly increase the risk of postoperative recurrence and shorten the survival rate of patients. Therefore, effective inhibition of tumor metastasis is the top priority of tumor therapy.

There are many key molecules involved in the adhesion, migration and invasion of cancer cells. Epithelial-mesenchymal transition (EMT) is the process by which epithelial cells transform into mesenchymal cells and then acquire the ability of migration and invasion. Moreover, matrix metalloproteinases (MMPs) can degrade the extracellular matrix, which is beneficial to tumor migration and invasion. DHA inhibited the migration and invasion of canine mammary cancer cells by regulating the EMT-related genes (Slug, ZEB1, ZEB2 and Twist) 40. In addition, DHA was shown to inhibit the proliferation and EMT of SGC7901 gastric cancer cells and downregulated the expression of Snail and PI3K/AKT signaling pathway, thereby inhibiting metastasis 41. Yuyuan Yao et al. found that DHA suppressed the activation of cancer-associated fibroblasts (CAFs) and mouse cancer-associated fibroblasts (L-929-CAFs) by inhibiting transforming growth factor-β (TGF-β signaling and reducing the interaction between tumor and tumor microenvironment. Therefore, DHA inactivated CAFs and inhibited cancer metastasis 42. By using an ovarian HO8910PM xenograft tumor model, DHA was shown to inhibit tumor metastasis *in vivo*. Interestingly, DHA inhibited the development of ovarian cancer by downregulating phosphorylated focal adhesion kinase (pFAK), MMP-2, von willebrand factor (vWF) and macrophage infiltration 43. A recent study showed that DHA inhibited HNSCC cell migration and invasion by blocking the phosphorylation of STAT3 and polarization of M2 macrophages 44. Urokinase-type plasminogen activator (uPA) is considered a marker of poor prognosis, and the expression level of uPA is positively correlated with relapse risk in breast cancer patients 39. Interestingly, we found that DHA could inhibit the growth and migration of breast cancer cells by inhibiting the expression of uPA 45.

Likewise, some signaling pathways were identified in the process of tumor metastasis inhibited by DHA. Yunli Tong et al. observed that DHA could inhibit cell proliferation, migration, invasion, cancer stem cells and EMT of non-small cell lung cancer (NSCLC). In these processes, the suppression of Wnt/β-catenin signaling pathway played an important role 46. Another study showed that DHA was able to inhibit the migration and invasion of NSCLC cells even at low concentrations. Besides, blocking NF-κB signaling could largely abolish the inhibition of GLUT1 transport to the plasma membrane by DHA 47. DHA also inhibited cell viability, migration, invasion and induced apoptosis of epithelial ovarian cancer (EOC) cells via inhibiting the hedgehog signaling pathway 48 (Figure 3). A recent study identified that DHA inhibited the growth and invasion of gastric cancer cells and confirmed that Cyclin D1-CDK4-Rb signaling played an important role in this process 49. In addition, DHA could inhibit the proliferation, migration and invasion of MDA-MB-231 breast cancer cells with the AKT/steroid receptor coactivator (SRC) signaling pathway involved, thereby inhibiting breast tumor-induced osteolysis 50. Interestingly, Juliano D. Paccez et al. identified that DHA acted as an Axl inhibitor in prostate cancer, blocking the expression of Axl through the miR-34a/miR-7/JARID2 pathway, thereby inhibiting the proliferation, migration and invasion of prostate cancer cells. These results provided a new molecular basis for the treatment of metastatic prostate cancer 51.

### Inhibition of angiogenesis

In the growth of solid tumor, if there is not enough supply of oxygen and nutrients, the growth of tumor will be limited to about 1-2 mm^3^ 52. Therefore, the continued growth and metastasis of tumor must depend on the angiogenesis. Inhibition of tumor angiogenesis has become an important anticancer therapy. DHA has been proved to have a significant effect on angiogenic activity. DHA significantly inhibited the proliferation, migration and tube formation of human umbilical vein endothelial cells (HUVECs) 53. Shuangjia Wang et al. found that DHA inhibited angiogenesis and tumor growth in pancreatic cancer cells, reduced NF-κB DNA binding activity, and downregulated the pro-angiogenic gene in downstream 54. Moreover, another study found that DHA-induced microRNA-mRNA regulatory networks promoted apoptosis and inhibited angiogenesis in pancreatic cancer cells, which were consistent with experiments results *in vivo* 55. Another study showed that DHA induced autophagy of HUVECs by inhibiting AKT/mTOR signaling 56. The study found that DHA enhanced vascular endothelial growth factor receptor 1 (VEGFR1) expression through upregulation of ETS-1. Therefore, the authors hypothesized that DHA's anti-angiogenic effect might be due to the inhibition of VEGFR2-mediated angiogenesis 57.

### Improvement of immunity

DHA has made great progress in the treatment of systemic lupus erythematosus mainly because of the inhibition of B lymphocytes. This reveals the potential role of DHA in immunotherapy. Shokoofe Noori et al. demonstrated that in a sheep red blood cell (sRBC) xenograft model, delayed-type hypersensitivity (DTH) was significantly increased in the treatment of DHA compared with that observed with the control regimen treatment. Besides, in a spontaneous mouse mammary tumor (SMMT) xenograft model, the level of IFN-γ was increased, while those of IL-4 and splenic CD4^+^CD25^+^Foxp3^+^ regulatory T lymphocytes were significantly reduced with the DHA treatment 58. Zhonghai Zhou et al. studied the effect of DHA on γδ T cells (a type of immune cells). The results revealed that an appropriate concentration of DHA was beneficial to the amplification of γδ T cells. Notably, DHA could promote the killing effect on pancreatic cancer cells by enhancing the proliferation of γδ T cells. In this process, the upregulation of some cytotoxic effector molecules (such as perforin and granzyme B) and IFN-γ would help to the killing efficacy on pancreatic cancer cells, which may be vital mechanisms for DHA to enhance the anti-tumor effect of pancreatic cancer cells 59. A study of DHA in melanoma showed that DHA induced the proliferation of IFN-γ^+^CD8^+^ T cells in tumor microenvironment and mouse spleens, while the number of CD4^+^CD25^+^Foxp3^+^ T cells and IL-10^+^CD4^+^CD25^+^ T cells returned to normal, thus enhancing the anti-tumor immunity of mice 60.

### Induction of autophagy

Autophagy is a kind of self-degradation process in the normal life of cells. Specifically, it means that some intracellular components of cell are wrapped in the membrane to form a cystic structure and transport to lysosome for degradation. There is an increasing evidence showed that the autophagy-mediated triggering of type II programmed cell death remarkably improved the therapeutic effects of DHA towards xenogeneic breast cancer 61. Boning Li et al. studied new targets for DHA in EOC cells and observed that DHA induced autophagy, while autophagy inhibitors (chloroquine and bafilomycin A) were able to reverse the cell growth inhibition and cycle arrest induced by DHA 62. Xinli Shi et al. described the relationship between inflammatory body and autophagy induced by DHA in HCC. They revealed that DHA promoted the AIM2/caspase-1 inflammasome, induced nuclear and mitochondrial DNA damage and ultimately promoted autophagy in HepG2215 cells 63. Cells treated with DHA exhibited the trait of autophagy with JNK pathway activated and Beclin 1 expression upregulated. Specifically, after treatment with DHA, the produced ROS led to JNK activation 64. Another study demonstrated that DHA inhibited the migration of esophageal cancer cells by inducing autophagy 65. These studies suggested that autophagy induction might be the key to the anticancer effect of DHA.

In addition, some clinical drugs can also kill tumor cells together with DHA by enhancing the level of autophagy. Rapamycin is an antibiotic that can induce autophagy in various cell types. One present study revealed that rapamycin significantly promoted the apoptosis of breast cancer cells induced by DHA, but this effect will be reduced following Atg7 knockdown. Meanwhile, rapamycin could promote the level of death‑associated protein kinase (DAPK) expression by increasing autophagy‑related 7 (Atg7) expression, and then together with DHA to enhance breast cancer cell apoptosis. Hence, this combined anticancer effect depended on the induction of autophagy 66.

### Induction of ER stress

ER is an organelle responsible for protein synthesis, folding and secretion in eukaryotic cells. However, ER homeostasis imbalance can lead to ER stress, and then affect the normal life of cells. Tumor cells contain more iron transporter receptors than normal cells, and iron can combine with the metabolites of DHA to form free radicals that lead to cytotoxic effects. Therefore, it is generally believed that excessive free iron ions in tumor cells are key factors for the ability of DHA to selectively kill tumor cells. Glucose regulatory protein 78 (GRP78, an ER stress-related molecule) was upregulated after DHA treatment. Further studies found that DHA increased the expression of GRP78 and DNA damage-inducing gene 153 (GADD153, another ER stress-related molecule). However, when HCT116 colon cancer cells were pre-treated with the iron chelator deferoxamine mesylate salt (DFO), the induction of GRP78 and GADD153 would be abolished. Thus, these results demonstrated that DHA-induced ER stress required iron 67. Controversially, another recent study showed that DHA dimer NSC735847 was cytotoxic for colorectal cancer. However, its induction of ER stress required the presence of heme, not the iron 68. This might be due to some chemical changes in DHA dimers compared with DHA monomers, so the mechanism of inducing ER stress might be changed.

## New methods to improve the clinical efficacy of DHA

DHA is a sesquiterpene lactone compound containing peroxide bridges. The unique structure of DHA results in poor water solubility and bioavailability, and multiple injections are often required to achieve the desired therapeutic effect, which greatly hinders the clinical application of DHA in cancer 69. To overcome this obstacle, there are many new approaches for DHA usage, such as the use of new drug carriers and polymers, new drug delivery systems and combination therapies. Here, we make a systematic summary of the reported improvements in the use of DHA.

### Drug carrier and polymer

Encapsulating DHA in carrier materials or designing it as a polymer may greatly improve the water solubility and bioavailability. Qian Sun et al. encapsulated DHA in gelatine (GEL) or hyaluronic acid (HA) nanoparticles using an electrostatic field system. The results revealed that DHA nanoaggregates exhibited higher anti-proliferative activity and bioavailability than DHA alone in A549 lung cancer cells. The reason may be that the hydrophilic GEL or HA nanoparticles have higher water dispersion after aggregation 70. Similarly, Lin Dai et al. designed a novel polymer-drug conjugate, multi-arm polyethylene glycol-DHA (PEG-DHA). They connected DHA with multi-arm polyethylene glycol. The PEG-DHA conjugate showed a moderate drug loading value, better water solubility (82-163-fold that of DHA), and a better anticancer effect *in vitro*. Subsequently, tumor xenograft assay demonstrated PEG-DHA had stronger efficacy on inhibiting tumor growth than native DHA in NSCLC. Owing to the excellent anticancer efficacy, high drug loading capacity, suitable molecular weight and minimal inherent impurities during conversion, 8armPEG40K-DHA was expected to undergo further clinical development for the treatment against NSCLC 71. Yingjie Hu et al. developed a combination therapy strategy by constructing nanostructured DHA and epirubicin liposomes. In their study, the results showed that DHA improved the inhibitory effect of epirubicin towards breast cancer cells *in vitro* and *in vivo*, and this combination induced type I and type II programmed death of breast cancer cells. Besides, the nanostructured DHA plus epirubicin liposomes lengthened the drug's circulation and promoted its accumulation in breast cancer tissues 72. There was a study explored the role of R_8_-modified epirubicin-dihydroartemisinin liposomes that targeted NSCLC cells. In this context, the liposomes disrupted vasculogenic mimicry (VM) channels and inhibited tumor metastasis. This strategy showed significant anti-tumor efficacy owing to increased selective accumulation of chemotherapeutic drugs at the tumor 73. Another study found that octreotide (OCT)-modified daunorubicin plus DHA liposomes had stronger anti-tumor effects than either treatment alone and could inhibit breast cancer invasion 74, which may provide new strategies for the treatment of metastasis breast cancer.

### Drug delivery system

The endoperoxide bridge structure of DHA is important for its antimalarial and anticancer activities, and DHA is thought to exert its cytotoxic effect through Fe (II)-mediated endoperoxide cleavage 75. Cancer cells often overexpress transferrin receptor (TfRs) to ingest iron, while TfR levels are almost undetectable in most normal cells 76. Therefore, Ikuhiko Nakase et al. made a series of artemisinin-tagged transferrin (ART-Tf) conjugates, which retained the activity of artemisinin. It was demonstrated that ART-Tf conjugates played a cytotoxic role through the mitochondrial pathway of apoptosis in prostate cancer cells 77. Thus, TfRs may be a biomarker for the selective delivery of anticancer drugs. Interestingly, Narendra et al. adopted a treatment method of “full transferrin+dihydroartemisinin”. This combination used an original approach in the treatment of breast cancer, where oral iron salts such as ferrous sulfate or ferrous citrate would increase the iron content in breast tumor, making cells more susceptible to cytotoxic effects. Thus, with the use of appropriate dosing regimens, oral or gastrointestinal administration of artemisinin analogues plus ferrous salts may be effective anti-breast cancer therapies 78. Further, a new study describes a hydrogel tumor treatment system that works in conjunction with DHA. Authors designed a hydrogel containing FeCl, traditional Chinese ink and agarose hydrogel. Heat caused the hydrogel to hydrolyse reversibly, and the iron then diffused from the hydrogel to the tumor microenvironment, where it was reduced to iron, broke the inner peroxides bridge of DHA and released free radicals that showed a powerful anticancer effect. Experiments have also shown that DHA-Fe has significant efficacy in chemodynamic therapy (CDT) and photodynamic therapy (PDT) 79.

## Combination medication

Combination of drugs is a common clinical cancer treatment method. The multi-drug resistance of tumor cells and adverse reactions to chemotherapy drugs often lead to the failure 80. Remarkably, the combination of drugs would enhance the anti-tumor effect of drugs and even reverse some known drug resistance 81. Current research showed that DHA had a stronger anti-tumor effect in the synergy with a variety of chemotherapeutic drugs, and DHA could reverse the drug resistance of certain cancer cell lines. Moreover, some new technologies have been used in cancer treatment combined with DHA.

### Improvement of anticancer effect

DHA in combination with some chemotherapeutic drugs can greatly enhance the anticancer effect. The combination of DHA and carboplatin (CBP) showed great inhibition on the growth of ovarian cancer cells as well as in ovarian A2780 and OVCAR-3 xenograft tumor models, which might be achieved through death receptors and the mitochondria-mediated caspase-dependent apoptosis pathway 82. Furthermore, Huijun Zhou et al. studied the effect of DHA in combination with cyclophosphamide (CTX) or cisplatin. They observed that DHA induced apoptosis in Lewis lung carcinoma (LLC) cells and affected the expression of VEGF receptor KDR/flk-1. Furthermore, in tumor xenograft models, compared with the drug alone, this combination showed stronger anti-growth and anti-metastasis effects. This study revealed the potential clinical significance of treating LLC using a combination DHA and chemotherapeutics (such as CTX and cisplatin 83. Gefitinib is a commonly used drug for the treatment of patients with local or metastatic NSCLC. Notably, DHA enhanced gefitinib-induced inhibition of NCI-H1975 lung cancer cells in migration and invasion via AKT/mammalian target of rapamycin (mTOR)/STAT3 signaling pathway, and co-administration of DHA and gefitinib induced cell cycle arrest in the G2/M phase 84 (Figure 3). Onconase (Onc) is a ribonuclease from leopard frog oocytes or early embryos and is used to treat malignant mesothelioma in clinical. A further study found that Onc combined with DHA had a strong antiangiogenic efficacy with low toxicity and immunogenicity, and this combination can serve as a novel regimen for NSCLC 85. DHA also cooperated with trastuzumab for the treatment of HER2/neu positive breast cancer to induce apoptosis of tumor cells 30. Notably, one study showed that DHA synergized with docetaxel, resulting in an enhanced anti-tumor efficacy and increased overall survival of xenografted mice 51. The combination of DHA and curcumin (Cur) exhibited synergistic anti-tumor effects on SKOV3 ovarian cells *in vitro* and *in vivo*. Furthermore, this combination could effectively weaken the expression of the oncogene midkine (MK) and upregulate miR-124 expression 86. DHA could inhibit the growth of HCC cells (HepG2, Hep3B, BEL-7404 and Huh-7) and pancreatic cancer cells (BxPC-3 and PANC-1). DHA combined with gemcitabine induced cell cycle arrest in G1 phase and promoted cell apoptosis. Specifically, DHA inhibited gemcitabine-induced NF-κB activation. Further, xenograft tumors *in vivo* also confirmed these results 87, 88.

Doxorubicin (DOX) is a commonly used anti-tumor drug, which can exert a strong cytotoxic effect by inhibiting the synthesis of DNA and RNA of cancer cells. DHA and DOX had been found to work together to inhibit the proliferation of MCF-7 breast cancer cells. Compared with monotherapy, the combined therapy significantly reduced mitochondrial membrane potential, activated the caspase cascade and finally induced apoptosis 89. Similarly, Maria Lucibello et al. also demonstrated the synergy between DHA and DOX. DHA could significantly enhance the anti-tumor effect of DOX on triple-negative breast cancer cells, thus leading to an increased induction of apoptosis 30. Furthermore, the combination of DHA and DOX was shown to promote apoptosis at optimal concentrations of 10 μg/ml of DHA and 10 μg/ml of DOX. The mouse HeLa tumor model showed that this combination significantly inhibited tumor growth without significant toxicity 90.

Remarkably, DHA in combination with several inhibitors has also shown a significant anti-tumor efficacy. DHA could directly target platelet-derived growth factor receptor-alpha (PDGFRα) to inhibit the growth and metastasis of ovarian cancer cells. When DHA was used in combination with PDGFRα inhibitors (sunitinib and sorafenib), it could sensitize ovarian cancer cells to PDGFR inhibitors and achieved effective therapeutic efficacy 91. Moreover, DHA combined with 2DG (a glycolysis inhibitor) synergistically induced apoptosis through both exogenous and endogenous apoptotic pathways 92. Chris Zhiyi Zhang et al. demonstrated that histone deacetylase inhibitors (HDACis) enhanced the anti-tumor effect of DHA by inducing apoptosis. For instance, the combination therapy reduced mitochondrial membrane potential compared with DHA treatment alone. In addition, DHA was observed to induce apoptosis, increase the expression of p53 and Bak, decrease the expression of Mcl-1 and pERK, and activate caspase 3 and poly ADP-ribose polymerase (PARP). The results demonstrated the synergistic effects of DHA and HDACi in HCC 93. Liping Wu et al. identified the synergistic anticancer effect with farnesylthiosalicylic acid (FTS, a RAS inhibitor) and DHA for the first time. FTS could sensitize HCC cells to DHA treatment by enhancing the intrinsic and extrinsic apoptotic pathways. Thus, this study gave a strategy for the clinical application of ARTs and FTS in the treatment of HCC 94. NSCLC cells typically harbour epidermal growth factor receptor (EGFR) or RAS mutations 95. Xiaohui Yan et al. found that DHA combined with ABT-263 (a Bcl-2 family inhibitor) could induce apoptosis of NSCLC harbouring EGFR or RAS mutations. Specifically, DHA effectively inhibited the phosphorylation of STAT3, and STAT3 inactivation resulted in the down-regulation of Mcl-1 and survivin, thereby enhancing the cytotoxicity induced by ABT-263. These data provided a new treatment strategy for treating NSCLC with EGFR or RAS mutations 96. Moreover, data presented by Qin He et al. indicated that DHA inhibited PI3K/AKT and extracellular signal-regulated kinase (ERK) pathways and activated the exogenous and endogenous cell death signals in prostate cancer cells. The combination treatment of DHA and tumor necrosis factor-related apoptosis inducing ligand (TRAIL) greatly improved cell killing. In summary, the authors propose new clinical approaches for prostate cancer therapies, namely, treatment with DHA alone or in combination with TRAIL 97.

Interestingly, DHA in combination with some new therapies may also promote anti-tumor effects. PDT is a photodynamic reaction between a photosensitizer and light of the corresponding wavelength, which results in the production of singlet oxygen, thereby killing tumor cells. PDT has been widely used in the treatment of esophageal cancer, and can significantly relieve esophageal obstruction and prolong survival in patients. However, PDT-induced NF-κB activation may lead to a decline in the efficacy of PDT therapy 98, 99. Yanjing Li et al. showed that DHA enhanced PDT-induced cell growth inhibition and apoptosis 100, and an additional investigation further reported that DHA increased the sensitivity of esophageal cancer cells to PDT by inhibiting the NF-κB/HIF-1α/VEGF pathway 101. Furthermore, CDT can also be combined with DHA. In a recent study, DHA was added to magnetic nanoparticles (MNP), and the MNP-DHA has shown an effect in the treatment of intractable breast cancer. These studies showed that blank MNP had almost no cytotoxicity, whereas MNP-DHA had a significant inhibitory effect on two invasive breast cancer cell lines (MDA-MB-231 and MDA-MB-453 cells). Under the acidic condition in the tumor microenvironment, MNP could produce ferrous ions and catalyse DHA to produce a large amount of ROS, thereby leading to cell death and greatly improving the therapeutic effect of CDT 102.

### Reversal of resistance

Cisplatin is one of the most commonly used chemotherapy drugs. Compared with that observed in control SKOV3 cells, mTOR phosphorylation was shown to be abnormally activated in cisplatin-resistant ovarian cancer cells (SKOV3/DDP) after cisplatin monotherapy. Further, the authors found that DHA promoted autophagy and apoptosis in SKOV3/DDP cells, and the inhibition of mTOR signaling pathway was involved. Notably, the inhibition of mTOR signaling pathway was involved in this process. Therefore, it was concluded that the killing effect of DHA towards SKOV3/DDP cells was achieved by inhibiting cisplatin-induced mTOR activation 103. Arsenic trioxide (As_2_O_3_, ATO) is a curative anticancer drug used to treat acute promyelocytic leukaemia and some specific cancers 104. However, lung cancer cells were recently observed to be resistant to ATO. Hongyu Chen et al. investigated the possibility of combining DHA with ATO. They found this combination synergistically promoted the apoptosis of A549 lung cancer cells by increasing the levels of ROS and DNA damage. More importantly, the combination therapy did not have significant adverse effects on normal human bronchial epithelial cells 105. Apo2 ligand (Apo2L)/TRAIL is considered as a prospective anticancer agent, but the chemical resistance affects its efficacy. It was reported that combination therapy with DHA and Apo2L/TRAIL promoted the induction of apoptosis in BxPC-3 and PANC-1 pancreatic cancer cells compared to monotherapy. In this study, DHA induced DR5 and regulated apoptosis-associated proteins to mediate cell apoptosis by producing ROS 106.

## Preliminary studies of clinical anticancer effects

### Preclinical trials

In addition to numerous basic experimental studies on the anticancer effects of DHA, there are also some preliminary researches on its pharmacodynamics and pharmacokinetics.

Since the binding affinity of DHA to serum proteins can affect the effectiveness of the drug, one study examined the binding of DHA to serum albumin under near-physiological conditions. The data showed changes in enthalpy and entropy, where hydrophobic force was the most important contributor. Interestingly, molecular docking studies revealed small changes in the chemical structure of a drug would significantly affect the binding of a drug to its target protein 107.

The cytochrome P450 (CYP) enzyme system can greatly influence the oxidative metabolism of drugs, and the modification of this system is an important factor influencing drug-drug interactions (DDIs). The effects of ARTs on seven major human liver CYP isoforms (CYP1A2, CYP2A6, CYP2B6, CYP2C9, CYP2C19, CYP2D6 and CYP3A4) were evaluated and DDI risk was predicted *in vivo*. In conclusion, DHA was shown to inhibit the enzyme activity of CYPs, mainly through mixed inhibition 108. The inhibition of CYP enzymes in the body may result in an increased drug plasma concentration, thereby leading to the occurrence of adverse reactions.

Animal models (such as cats and dogs) can also be used to study preclinical problems prior to clinical studies. A large clinical study of more than 15,000 malaria patients found no significant toxicity of DHA 109, 110. However, preclinical toxicity studies in healthy experimental dogs using artemisinin derivatives showed neurotoxicity 111. Similarly, canines with spontaneous tumors showed good tolerance to artemisinin but had anorexia and low bioavailability after oral administration 112. Taken together, these results indicate that ARTs may have potential toxicity.

### Clinical trials

Previous studies have confirmed that DHA is cytotoxic to cervical cancer cells. Jansen et al. further investigated the clinical effect and safety of oral Artenimol-R (the succinate ester of DHA) in patients with advanced cervical cancer. Ten patients were treated with DHA for 28 days, with follow-up monitoring of clinical symptoms, vaginal discharge, pain and adverse reactions. Furthermore, the expressions of tumor-related markers in the biopsy samples were analysed by immunohistochemistry. Overall, the results showed that DHA treatment induced clinical symptom relief and the median time to symptom disappearance decreased compared with that observed in the control group. Grade 3 or 4 adverse events did not happen. Besides, the expressions of some proliferation markers such as p53, EGFR and Ki-67 were reduced; the number of blood vessels stained with CD31 was decreased; and the expression of transferrin receptor protein 1 (CD71) was increased. Thus, this study provided a basis that DHA could improve the clinical symptoms of advanced cervical cancer patients and it was well tolerated 113. However, it is necessary to conduct further survival trials in patients with advanced cervical cancer.

We have reviewed the clinical trial websites of various countries worldwide. Thus far, in addition to anti-malaria, we have identified only three clinical trials of DHA in *Clinicaltrials.gov* and *Chinadrugtrials.org.cn*. There is only one clinical trial on anti-tumor effect of DHA named *Icotinib Combined With DHA Therapy in Patients With Advanced NSCLC*. This phase II clinical study explored the anti-tumor effect of the combination of DHA and icotinib on EGFR-positive NSCLC patients, with identification number NCT03402464. The method of medication is as follows: On day 1-3, oral DHA was given at 20mg per day, the dose of DHA is increased to 40mg daily on day 4 to 6, and 80mg twice daily until disease progressed or intolerable toxicity. This clinical trial is still ongoing. The other two clinical trials are phase II clinical trials for the treatment of systemic lupus erythematosus in China, with identification numbers CTR20171440 and NCT03396393, respectively.

Due to the limited anti-tumor clinical trials of DHA, we also reviewed some clinical trials of ARTs, and there have been some anti-tumor clinical trials on artesunate. The treatment of metastatic breast cancer with artesunate was proved to be well tolerated by patients 114. Moreover, artesunate showed a good therapeutic effect on colorectal cancer 115. The pharmacokinetic analysis of oral artesunate demonstrated that the apparent clearance rate of DHA (the active metabolite of artesunate) increased with time, and the drug could be stably metabolized 116. Remarkably, treatment of metastatic breast cancer with artesunate showed ototoxicity 117, which also needs to be noticed in clinical trials of DHA.

Given the results discussed above, artesunate has been proven to have significant anti-tumor effects in clinical trials, and it can be speculated that DHA (its active metabolite) has great potential in cancer treatment. However, the anti-tumor research of DHA remains nascent and incomplete. Hence, we need to conduct further research to develop chronic toxicology and drug interaction studies of DHA, as well as more and larger clinical trials. These studies will lay the foundation for the clinical use of DHA as an anticancer drug and provide a more convincing evidence for the applicability of DHA in clinical oncology.

## Current developments and limitations of DHA

DHA is a commonly used antimalarial drug with anticancer effects, and it is receiving more and more attention. In the research and development of drugs, exploring new indications using old drugs will greatly shorten the time of drug development. As an antimalarial drug, the dose for adults is 120 mg on the first day, and 60 mg per day thereafter for 5-7 days. Within the normal dosage, only a small number of patients will experience a mild transient reduction in reticulocytes, and there is no serious adverse reaction. Therefore, the known drug safety makes the development of new indications for DHA convenient and efficient.

In the past two decades, numerous reports have emerged on the anti-tumor activity of DHA. These findings strongly supported that DHA had effective anticancer roles, which indicated that DHA could be a potential candidate for cancer treatment. Table 1 lists the range of effective doses and effects of DHA used in various cancer types, which provides a comparison and reference of DHA in various disease models.

Recently, cancer treatment has focused on individualized medications and combined drug therapy to improve drug sensitivity and therapeutic effects 118. According to the research above, DHA combined with a variety of existing chemotherapy drugs have demonstrated significant anti-tumor effects, which could be helpful to solve current clinical drug resistance of tumor. This review summarizes the combination of DHA and chemotherapy drugs, and shows the effective doses and effects of DHA *in vitro* and* in vivo*, thereby providing a reference for future combined applications of clinical drugs (Table 2).

However, there are still some limitations regarding anticancer studies of DHA. Firstly, the anticancer mechanism of DHA is still unclear and requires further research. Secondly, the metabolites and metabolic pathways of DHA *in vivo* have not been clearly studied, which will help us better understand the mechanism of DHA *in vivo*. Thirdly, the neurotoxicity and ototoxicity observed in preclinical trials cannot be ignored, thus, new drug dosages or improved drug structures should be developed in the future to avoid DHA-mediated toxicity and side effects. Furthermore, since DHA has limited clinical trials in tumor, its clinical anticancer efficacy and drug resistance remain unknown.

## Conclusion

The research of anticancer effect of DHA is promising. Evidence suggests that DHA has powerful anti-tumor efficacy. This review systematically summarized the pharmacological effects and potential molecular mechanisms of DHA *in vitro* and *in vivo*. Furthermore, new methods to improve the water solubility and bioavailability of DHA as well as the combination medication were also discussed which highlighted the application prospects of DHA as an anticancer drug. Moreover, clinical trials further demonstrated the powerful anti-tumor effect of DHA. It is worth noting that, the molecular mechanism of DHA against tumors is not comprehensive and unsolved potential toxicity questions are notable deficiencies. Moreover, bioinformatics and experimental analysis of drug interactions are also needed in order to better understand the role of DHA in the body. Interestingly, there is evidence that DHA has potential efficacy on immune cells in the tumor microenvironment, and DHA might be considered in combination with immunotherapy in the future. Comprehensive research is required so that DHA can become a highly effective and low toxicity anticancer drug for the benefit of patients.

## Figures and Tables

**Table 1:** Summary of the anticancer data about effective dose ranges and effects of dihydroartemisinin *in vitro* and *in vivo*

Cancer type	Model type	Effective doses	Main effects	Reference
Lung cancer	A549 cell	10-30μM	Inhibited cell proliferation Induced cell cycle arrest Upregulated the ratio of Bax/Bcl-2 Activated of caspase 3 and cytochrome c Inhibited the AKT/GSK3β/cyclin D1 pathway	12
	PC-14 cell	18μM	Inhibited cell proliferation Induced apoptosis Induced Ca^2+^ increasing Increased p38 phosphorylation	28
	A549, H1299 cells	7.5-30μM	Inhibited cell proliferation Induced G1 cell cycle arrest by inhibiting cyclin D1 Inhibited cell migration and invasion Suppressed EMT Inhibited Wnt/β-catenin signaling pathway	46
	A549 tumor xenograft model	60 mg/kg/5 times/week for 4 weeks (i.g.)	Inhibited tumor growth Downregulated Wnt5-a/b, LRP6, and Dvl2 Upregulated NKD2 and Axin2	46
	A549, H1975 cells	7.5-30μM	Inhibited cell viability, migration and invasion Decreased ATP production, glucose uptake and lactate levels Inhibited transcriptional activity of the NF-κB Inhibited plasma membrane location of Glut 1	47
	A549 tumor xenograft model	50 or 100 mg/kg/day for 28 days	Inhibited tumor metastasis	47
	A549 cell	GEL/DHA and HA/DHA aggregates10-40 μg/mL	Inhibited cell proliferation Induced apoptosis	70
	LLC tumor xenograft model	DHA or PEG-DHA (i.v.)a. 50 mg/kg single dose b.10 mg/kg, every 2 days, q2d×5	Inhibited tumor growth	71
	LLC cell	5-160μM	Inhibited cell proliferation Induced apoptosis Downregulated KDR/flk-1 in mRNA and protein level	83
	NCI-H1975 cell	5-100μM	Inhibited cell proliferation Induced apoptosis	84
	A549, PC-9 cells	10-64μM	Inhibited cell viability and colony formation Induced apoptosis Inhibited the glucose uptake Decreased ATP and lactate levels Downregulated p-mTOR activation and GLUT1 expression Increased ROS production	92
	H1975, HCC827, H1650, H3255A549, H727,H1299,MV522 cells	2.5-20μM	Enhanced ABT-263-induced apoptosis Induced apoptosis Downregulated Mcl-1, survivin Suppressed STAT3 phosphorylation	96
	H1975 tumor xenograft model	25 mg/kg/day for 15 days (i.g.)	Inhibited tumor growth	96
	A549 cell	15-90μM	Inhibited cell proliferation Decreased colony formation Induced DNA damage Enhanced the intracellular ROS levels Induced cell cycle arrest at G2/M phase Induced apoptosis	105
Breast cancer	T-47D cell	20-60μM	Inhibited cell proliferation Induced apoptosis Upregulated caspase-8 and cleaved caspase-9; activated Bid and Bim Downregulated Bcl-2 Released cytochrome c from mitochondria into the cytosol	20
	MDA-MB-231, MCF7, SKBR3, BT-474 cells	20, 50μM	Downregulated TCTP and phospho-TCTP in protein level TCTP knocking down in MDA cells enhanced the cytotoxicity of DHA	30
	CHMm cell	5-20μM	Inhibited cell proliferation Inhibited TGF-β1-induced migration and invasion Downregulated Slug, ZEB1, ZEB2 and Twist in mRNA level	40
	4T1, CAFs, L-929-CAFs cells	30, 50μM	Inhibited CAFs and L-929-CAFs activation Suppressed TGF-β pathway	42
	4T1-luciferase cells tumor xenograft model; 4T1-luciferase cells plus L-929 cells tumor xenograft model	100 mg/kg/day for 28 days (i.g.)	Suppressed CAFs-induced cancer growth and metastasis Suppressed TGF-β signaling	42
	MDA-MB-231, MCF-7 cells	6.25-100μM	Inhibited cell proliferation Inhibited cell migration	45
	MDA-MB-231 cell	1.56-6.25μM	Suppressed AKT/SRC signaling pathway Inhibited cell proliferation, migration and invasion	50
	a. Te calvarial osteolysis mice model b. MDA-MB-231 tumor xenograft model	a. 50 and 100 μg/kg/day for 10 daysb. 100 μg/kg every other day for 28 days (i.p.)	a. Suppressed titanium-particle-induced osteolysis b. Inhibited breast cancer bone metastasis and osteolysis	50
	RIN cell	25-50μM	Inhibited cell proliferation	58
	a. sRBC tumor xenograft model b. SMMT	a. 1.95-4.85 μg/mouse/day for 24, 48 and 72h (footpad injection) b. 4.85 μg/mouse/day for 6 days (i.p.)	a. Increased in DTH response b. Inhibited tumor volume; increased lymphocyte proliferation index; upregulated IFN-γ; downregulated IL-4; decreased splenic CD4^+^CD25^+^Foxp3^+^T regulatory lymphocytes	58
	HTB 27 cell	200μM	Inhibited cell proliferation	78
Prostate cancer	PC‐3 cell	25-200μM	Inhibited cell proliferation Downregulated HSP70 in mRNA and protein level Upregulated Apaf‐1, caspase‐3, AIF protein expression	32
	LNCaP cell	10-160μM	Inhibited cell proliferation Downregulated HIF-1α by PI3K/AKT pathway	34
	C4, C4-2, C4-2B, DU145, PC-3, LNCaP cells	5μM	Inhibited cell proliferation Inhibited migration and invasion Inhibited Axl expression by inducing miR-7 and miR-34a expression	51
	DU145 tumor xenograft model	40 mg/kg/day for 50 days (i.p.)	Inhibited tumor growth Downregulated IL-6	51
	PC-3, DU145 cells	100μM	Induced apoptosis	77
	DU145, PC3, LNCaP cells	50μM	Inhibited cell proliferation Induced caspases 3, 8 and 9 activation Suppressed Akt phosphorylation Inhibited ERK1/ERK2 phosphorylation Upregulated DR5 in protein Increased DR5 promoter activity	97
Ovarian cancer	OVCA-420, OVCA-432, SKOV3, OVCAR-3 cells	2-20μM	Induced apoptosis and cell cycle arrest at G2 phase Decreased Bcl-x_L_ and Bcl-2 Increased Bax and Bad	29
	HO8910PM cell	12.5-50µM	Inhibited cell proliferation, adhesion, migration and invasion Downregulated pFAK, MMP-2, vWF and macrophage infiltration	43
	HO8910PM tumor xenograft model	50 mg/kg three times a week for 4 weeks (i.p.)	Inhibited tumor metastasis	43
	SKOV3, SKOV3‐IP, HO8910, HO8910‐PM cells	20-160μM	Inhibited cell proliferation, migration, and invasion Induced apoptosis Inhibited the hedgehog signaling pathway	48
	Hela, JAR, RD, HO-8910 cells	2.5-50μM	Inhibited cell proliferation Inhibited proliferation, migration and tube formation of HUVECs	53
	EOC, SKOV3 cells	30µM, 50µM	Inhibited cell proliferation Induced cell cycle arrest at G2/M phase Induced autophagy Suppressed NF-κB signaling pathway	62
	A2780, OVCAR-3 cells	5-50μM	Inhibited cell proliferation Induced apoptosis Released cytochrome c from the mitochondria to the cytosol Upregulated fas, FADD, Bax, cleaved-PARP Downregulated Bcl-2 and Bid	82
	A2780 and OVCAR-3 tumor xenograft model	10 and 25 mg/kg/5 days/week for 3 weeks (i.p.)	Inhibited tumor growth Downregulated the ratio of Bcl-2/Bax and p-caspase-8	82
	SKOV3 cell	2.5-80μM	Inhibited cell proliferation Induced cell cycle arrest at S and G2/M phase Induced apoptosis Downregulated MK levels of mRNA and protein	86
	SKOV3 cell xenograft tumor	30 mg/kg every two days for 5 weeks (i.p.)	Inhibited cell proliferation	86
	HeLa, OVCAR-3, MCF-7, PC-3, A549 cells	0.5-20μM	Inhibited cell proliferation Induced apoptosis	90
	HeLa tumor xenograft model	15 mg/kg for 35 days (intratumoral injection)	Inhibited tumor growth	90
	OVCAR3, A2780, SKOV3, OVCAR5 cells	5-25μM	Inhibited cell proliferation Induced apoptosis Downregulated PDGFRα expression Repressed EMT phenotype Inhibited cell migration Inhibited PI3K/AKT and MAPK pathways	91
	A2780 cell xenograft tumor	10 or 25 mg/kg, 5 days/week for 4 weeks (i.p.)	Inhibited cell tumor growth and migration Downregulated PDGFRα expression Repressed EMT phenotype Inhibited PI3K/AKT and MAPK pathways	91
	SKOV3, SKOV3/DDP cells	10-80μM	Induced cell cycle arrest and autophagy Downregulated c-Myc, cyclin A and p-mTOR activity	103
Esophageal cancer	Eca109, Ec9706 cells	2.5-120μM	Inhibited cell proliferation Induced apoptosis Induced cell cycle arrest at G0/G1 phase Upregulated Bax, procaspase-9 and LC3-IIDownregulated Bcl-2, Bcl-x_L_, procaspase-3, Cyclin E, CDK2 and CDK4	11
	Eca109 tumor xenograft model Ec9706 tumor xenograft model	2, 10, or 50 mg/kg/day for 2 weeks (i.p.)	Inhibited tumor growth Inhibited cell proliferation Induced apoptosis	14
	Eca109, Ec9706 cells	10-120μM	Inhibited glycolysis	35
	Eca109, Ec9706 cells	2.5-120μM	Inhibited cell proliferation Augmented PDT-induced growth inhibition Sensitized to PDT-induced apoptosis Downregulated Bcl-2, NF-κB and its downstream gene expression Upregulated Bax, p-caspase-3 and p-caspase-9	101
Hepatocellular cancer	HepG2, PLC/PRF/5, Hep3B cells	2.5-80μM	Inhibited cell proliferation and colony formation assay Induced apoptosis and G2/M arrest Released of cytochrome c Downregulated Mcl-1 Upregulated Bak	13
	HepG2 tumor xenograft model	20 mg/kg five times a week for 4 weeks (i.p.)	Inhibited tumor growth Upregulated Bak and cleaved caspase 3 Downregulated Mcl-1	13
	HepG2, 7402, LM3 cells	5-40μM	Inhibited cell proliferation Downregulated ROS	17
	HepG2, Huh-7, LO2, Hep3B cells	10-150μM	Inhibited cell proliferation Induced apoptotic Upregulated p-caspase-8, -9 and -3, Bax, Bak and Bim Downregulated Mcl-1	21
	SK-Hep-1 cell	20-80μM	Inhibited cell proliferation Induced caspase-dependent apoptosis Inhibited Sp1 pathway Suppressed MAPK pathways	22
	HepG2215 cell	5-40μM	Inhibited cell proliferation Induced autophagy Upregulated p-caspase-1, AIM2 inflammasome and intracellular ROS Inhibited cell movement capacity	63
	HepG2, Hep3B, BEL-7404, Huh-7 cells	1-50μM	Inhibited cell proliferation Induced apoptosis and G1-phase cell cycle arrest Downregulated cyclins and CDKs Upregulated Cip1/p21 and Kip1/p27 Upregulated ratio of Bax/Bcl-2, p-caspase-3	87
	HepG2, Hep3B tumor xenograft models	50 or 100 mg/kg/day5 days/week for 4 weeks (i.p.)	Inhibited tumor growth Downregulated Cyclin D1, Cyclin E, Cdk2, Cdk4 and E2F1 Upregulated p21, p27, p-caspase-3, cleaved PARP, Rb p53, the ratio of Bax/Bcl-2 and MDM2	93
	HepG2, PLC/PRF/5 cells	10μM	Upregulated MAP kinases Downregulated ERK	93
Gastric cancer	SGC-7901, BGC823, MGC803 cells	2.5-80μM	Inhibited cell proliferation Induced apoptosis, G1 cell cycle arrest and senescence Downregulated Bcl-2 Inhibited the migration and invasion Upregulated miR-15b and miR-16	10
	SGC-7901 tumor xenograft model	15 and 30 mg/kg/day for 21 days (i.p.)	Inhibited tumor growth Downregulated Ki-67 and Bcl-2 Upregulated miR-15b and miR-16	10
	SGC-7901 cell	1.25-20μM	Inhibited the growth of H. pylori and gastric cancer cells Suppressed H. pylori adhesion to gastric cancer cells Downregulated ROS production Inhibited the NF-κB signaling pathway	18
	MNU and H. pylori-induced gastric carcinogenesis mouse model	60 mg/kg, 3 times/week for 6 cycles	Inhibited the incidence of tumor modelsDownregulated IL-6, TNF-α IL-1β, COX-2 and p-IκBα	18
	BGC-823 cell	10-40μM	Inhibited cell proliferation Induced apoptosis Activated JNK1/2 and p38 MAPK signaling pathways	27
	BGC-823 tumor xenograft model	15 and 30 mg/kg/day for 21 days (i.p.)	Inhibited tumor growth	27
Pancreatic cancer	BxPC-3, AsPC-1 cells	5-80μM	Inhibited cell proliferation Downregulated cyclin D1 and PCNA Upregulated p21^WAF1/CIP1^ Induced apoptosis	25
	BxPC-3 tumor xenograft model	2, 10, and 50 mg/kg/day for 18 days (i.p.)	Inhibited tumor growth Downregulated Ki-67 Induced apoptosis	25
	BxPC-3, AsPC-1 cells	5-20μM	Induced cell cycle arrest at G0/G1 phase Downregulated of Cyclin E, CDK2, CDK4 and CDK6 Inhibited translocation Inhibited DNA-binding activity of NF-κB/p65	26
	BxPC-3, PANC-1 cells	12.5-100μM	Inhibited cell proliferation Inhibited NF-κB activation	54
	BxPC-3 tumor xenograft model	2, 10, or 50 mg/kg/day for 21 days (i.p.)	Inhibited tumor growth Inhibited NF-κB activation Downregulated VEGF, COX-2 and MMP-9	54
	PANC-1, BxPC-3 SW1990, CFPAC-1 cells	50μM	Upregulated miR-34a-5p, miR-195-5p, miR-30c-5p and miR-130b-3p Downregulated Cdk4, Cdk6, VEGF, IKKα, MEK1,E2F3, Rac1, E2F1, and CDC42 in mRNA level	55
	BxPC-3 tumor xenograft model	10 mg/kg/day for 18 days (i.p.)	Inhibited tumor growth Inhibited angiogenesis Induced apoptosis	55
	SW1990, BxPC-3, PANC-1 cells	12.5-200μM	Upregulated perforin, granzyme B, IFN-γ	59
	BxPC-3, PANC-1 cells	12.5-50μM	Inhibited cell proliferation Induced caspase-3-dependent cell death and autophagy Downregulated p-caspase-3 Upregulated JNK and beclin 1	64
Colorectal cancer	SW 948 cell	10-50μM	Inhibited cell proliferation Induced apoptosis Upregulated PPARγ, MMP-2 and -9 Inhibited cell migration	15
	AOM/DSS mice model	20 mg/kg/day for 30 days (i.p.)	Inhibited tumor growth and inflammation	15
	HCT116 cell	12.5-100μM	Inhibited cell proliferation Induced apoptosis Upregulated Bax, p-caspase-3/9, p-JNK1/2 and p38 MAPK Downregulated PARP, p-ERK1/2, p-JAK2 and p-STAT3	23
	HCT15, Colo205, HCT116 cells	10-80μM	Inhibited cell proliferation Induced apoptosis and necrotic cell death Induced ROS production Upregulated Mcl-1	36
	HCT116 cell	0.3-30μM	Inhibited cell proliferation, cell cycle arrest and apoptosis Upregulated GRP78 and GADD153 in mRNA levels Induced ER stress	67

i.p., Intraperitoneal; i.v., Intravenous; i.g., Intragastric

**Table 2:** Summary of the anticancer data about dose and effects of combination treatment with dihydroartemisinin *in vitro* and *in vivo.*

Cancer type	Model type	Combination	Effective doses	Main effects	Reference
Lung cancer	A549 cell	Epirubicin	Epirubicin: 0-5µM DHA: 0-50µM	Inhibited migration, invasion and VM channels Inhibited cell adhesion Induced apoptosis	73
	A549 cell	Epirubicin	R_8_ modified epirubicin-dihydroartemisinin liposomes epirubicin: 10µM epirubicin/DHA=1:5, molar ratio	Inhibited migration, invasion and VM channels Inhibited cell adhesion Induced apoptosis	73
	A549 tumor xenograft model	Cisplatin	Cisplatin: 2mg/kg/day (i.p.) DHA: 50, 100, 200 mg/kg/day (i.p.) every other day for five times for 25 days	Inhibited tumor growth Inhibited pulmonary metastasis	83
	LLC tumor xenograft model	CTX	CTX: 50 mg/kg/day (i.p.) DHA: 50, 100,200 mg/kg/day (i.p.) every other day for five times for 25 days	Inhibited tumor growth Inhibited pulmonary metastasis	83
	NCI-H1975 cell	Gefitinib	DHA: 10µM Gefitinib: 10µM	Inhibited cell proliferation Induced apoptosis, cell cycle arrest in G2/M phase Inhibited migration and invasion Downregulated cyclin B1, Cdk1, p-Akt, p-mTOR, p-STAT3 and Bcl-2 Upregulated Bax	84
	NCI-H661, SK-MES-1, SPC-A-1, A549 cells	Onc	Onc: 5µM DHA: 2.5µM	Inhibited cell proliferation Inhibited of endothelial cell tube formation	85
	A549 tumor xenograft model	Onc	Onc: 3 mg/kg (i.v.) Followed by DHA 10 mg/kg next day twice a week for 3 weeks (i.p.)	Inhibited tumor growth Inhibited tumor angiogenesis	85
	A549, PC-9 cells	2DG	2DG: 0.5-6mM DHA: 5-60μM	Inhibited glycolysis Induced apoptosis Upregulated cytochrome c and AIF cytoplasm	92
	H1975 tumor xenograft model	ABT-263	ABT-263: 100 mg/kg/day (i.g.)DHA: 25 mg/kg/day for 15 days (i.g.)	Inhibited tumor growth	96
	H1975, HCC827, H1650, H3255, A549, H727, H1299, MV522 cells	ABT-263	ABT-263: 2µM DHA: 15µM	Induced apoptosis Downregulated Mcl-1, survivin, p-STAT3	96
	A549 cell	ATO	ATO: 10μMDHA: 45μM	Inhibited cell proliferation Induced DNA damage Induced cell cycle arrest at G2/M phase Increased the intracellular ROS Induced apoptosis	105
Breast cancer	MDA, SKBR3 cells	DOXCisplatin	DOX: 1-5μM or Cisplatin: 50μM DHA: 50μM	Inhibited cell proliferation Induced apoptosis	30
	SKBR3 cell	Trastuzumab	Trastuzumab: 50 µg/ml DHA: 20μM	Induced apoptosis	30
	MDA-MB-231 cell	Rapamycin	Rapamycin: 100nM DHA: 10 µg/ml	Inhibited cell proliferation Induced apoptosis Upregulated Atg7 and DAPK mRNA levels	66
	MDA-MB-435S tumor xenograft model	Epirubicin	DHA plus epirubicin liposomes 4 mg/kg for both drugs once (tail vein injection)	Inhibited cell proliferation	72
	MDA-MB-435S, MDA-MB-231, MCF-7 cells	Epirubicin	a. DHA 10μM; epirubicin 5μM (free) b. DHA 10μM; epirubicin 5μM (liposomes)	Inhibited cell proliferation Induced autophagy and apoptosis Upregulated caspase-9, caspase-3, Bax, Beclin 1, LC3B and ROS Downregulated Bcl-2	72
	MDA-MB-435S, MCF-7 cells	Daunorubicin	a. Daunorubicin: 0-2.5μM DHA: 2.5, 5 or 25μM (free)b. Daunorubicin plus DHA liposomes (0-10μM)c. OCT-modified daunorubicin plus DHA liposomes	Inhibited cell proliferation Inhibited migration Downregulated α5β1-integrin, TGF-β1, VEGF, MMP2 and MMP9	74
	MDA-MB-435S tumor xenograft model	Daunorubicin	a. Daunorubicin plus DHA liposomesb. OCT-modified daunorubicin plus DHA liposomes5 mg/kg daunorubicin (tail vein injection)	Increased drug accumulation at tumor sites Inhibited tumor growth	74
	HTB 27	Holotransferrin	Holotransferrin: 1 mg/ml DHA: 200μM for 8 and 24hrs	Inhibited cell proliferation	78
	MCF-7, MDA-MB-231, T-47D cells	DOX	DOX: 1.25-20µM DHA: 2.5-40µM	Inhibited cell proliferation Induced apoptosis	89
Prostate cancer	DU145 cell	Docetaxel	Docetaxel: 2.4-625nM for 24 h DHA: 0.0048-5µM	Inhibited cell proliferation	51
	DU145 tumor xenograft model	Docetaxel	Docetaxel: 15 mg/kg on day 1, 8, 15, 38, 45, 52 (i.p.) DHA: 40 mg/kg/day (i.p.)	Inhibited tumor growth Downregulated IL-6 Prolonged survival	51
	DU145, PC3, LNCaP cells	TRAIL	TRAIL: 10-20 ng/ml DHA: 10-30µM	Inhibited cell proliferation Upregulated pro-caspases 8, 9 and 3 Induced apoptosis	97
Ovarian cancer	a. A2780, OVCAR-3 cells b. A2780 and OVCAR-3 tumor xenograft models	CBP	a. CBP 10µM plus 1µM DHA for 0, 24, 48 or 72hrs 500µM CBP plus 1µM DHA for 24hrsb. CBP: 120 mg/kg, once on day 0DHA: 25 mg/kg/5 days/week for 3 weeks (i.p.)	Induced apoptosis Inhibited tumor growth Downregulated the Bcl-2/Bax ratio and pro-caspase-8	82
	SKOV3 tumor xenograft model	Cur	Cur: 20 mg/kg (i.p.) DHA: 30 mg/kg (i.p.)every two days for 5 weeks	Inhibited tumor growth	86
	SKOV3 cell	Cur	Cur: 10μM DHA: 20μM	Inhibited cell proliferation Induced apoptosis and cell cycle arrest at S and G2/M Downregulated Bcl-2, MK levels of mRNA and protein	86
	HeLa, OVCAR-3, MCF-7, PC-3, A549 cells	DOX	DOX: 10 µg/ml DHA: 10 µg/ml	Induced apoptosis	90
	HeLa tumor xenograft model	DOX	DOX: 15 mg/kg/day DHA: 15 mg/kg/dayfor 35 days (intratumoral injection)	Inhibited tumor growth	90
	A2780 tumor xenograft model	Sorafenib	Sorafenib: 30mg/kg/day (i.g.) DHA: 30mg/kg/day (i.p.)5 days/week, for 4 weeks	Inhibited tumor growth and metastasis Inhibited PI3K/AKT and MAPK pathways Downregulated PDGFRα	91
	SKOV3, SKOV3/DDP cells	Cisplatin	Cisplatin: 20µMDHA: 40µM	Downregulated c-Myc, cyclin A and Bcl-2 Upregulated Bax and cleaved-caspase-3	103
Pancreatic cancer	BxPC-3 and PANC-1 cells	Gemcitabine	Gemcitabine: 50-500nMDHA: 50μM	Inhibited cell proliferation Induced apoptosis Abrogated gemcitabine-induced NF-κB Downregulated c-Myc, Cyclin D1, Bcl-x_L_ and Bcl-2 Upregulated Bax and pro-caspase-3	88
	BxPC-3 tumor xenograft model	Gemcitabine	Gemcitabine: 100 mg/kg/twice weekly (i.p.) DHA: 10 mg/kg/day for 21 days (i.p.)	Inhibited tumor growth Suppressed NF-κB DNA-binding activity Downregulated Ki-67	88
	BxPC-3 and PANC-1 cells	Apo2L/TRAIL	Apo2L/TRAIL: 50-200 ng/ml DHA: 25-100μM	Inhibited cell proliferation Induced apoptosis Upregulated Bax, caspase-3, caspase-8, caspase-9 and DR5 Downregulated Bcl-2	106
	BxPC-3 tumor xenograft model	Apo2L/TRAIL	Apo2L/TRAIL: 50μg/day for 21 days (i.p.) DHA: 10 mg/kg/day (i.p.)	Inhibited tumor growth	106
Colorectal cancer	HCT116 tumor xenograft model	Rac1 siRNA	Rac1 siRNA: 2 nmol/day for 2 days (intratumoral injection) DHA :10 mg/kg/day for 21 days (i.p.)	Inhibited tumor growth Reduced NF-κB DNA binding activity Upregulated p21, cleaved-caspase-3 and cleaved-PARP Downregulated PCNA, cyclin D1 and CDK4	16
	HCT116 and RKO cells	Rac1 siRNA	DHA: 10-50μM	Inhibited cell proliferation Induced apoptosis and cell cycle arrest at G1 phase Upregulated cleaved-caspase-3 and cleaved-PARP Inhibited cell migration Inhibited NF-κB mediated transcription	16

i.p., Intraperitoneal; i.v., Intravenous; i.g., Intragastric

## Abbreviations

AKT: the protein kinase B
Apo2L: Apo2 ligand
ARTs: artemisinin and its derivatives
Atg7: autophagy‑related 7
ATO: arsenic trioxide
CAFs: cancer-associated fibroblasts
CBP: carboplatin
CD71: transferrin receptor protein 1
CDT: chemodynamic therapy
CTX: cyclophosphamide
Cur: curcumin
CYP: cytochrome P450
DAPK: death‑associated protein kinase
DDI: drug-drug interaction
DFO: deferoxamine mesylate salt
DHA: dihydroartemisinin
DOX: doxorubicin
DTH: delayed type hypersensitivity
EGFR: epidermal growth factor receptor
EMT: epithelial-mesenchymal transition
EOC: epithelial ovarian cancer
ER stress: endoplasmic reticulum stress
ERK: extracellular signal-regulated kinase
FTS: farnesylthiosalicylic acid
GADD153: DNA damage-inducing gene 153
GEL: gelatine
GLS1: glutaminase
GLUT1: glucose transporter-1
GRP78: Glucose regulatory protein 78
Gsk3β: glycogen synthase kinase-3β
H. pylori: Helicobacter pylori
HA: hyaluronic acid
HCC: hepatocellular carcinoma
HDACis: histone deacetylase inhibitors
HIF-1α: hypoxia inducible factor-1α
HSP70: heat-shock protein 70
HUVECs: human umbilical vein endothelial cells
JAK2: janus kinase 2
JNK1/2: c-Jun NH_2_-terminal kinases
LLC: Lewis lung carcinoma
MK: midkine
MMPs: matrix metalloproteinases
MNP: magnetic nanoparticles
mTOR: mammalian target of rapamycin
NF-κB: nuclear factor kappa B
NSCLC: non-small cell lung cancer
OCT: octreotide
Onc: Onconase
p38 MAPK: p38 mitogen-activated protein kinase
PARP: poly ADP-ribose polymerase
PDGFRα: platelet-derived growth factor receptor-alpha
PDT: photodynamic therapy
PEG-DHA: polyethylene glycol-dihydroartemisinin
pFAK: phosphorylated focal adhesion kinase
PI3K: phosphatidyl-inositol-3-kinase
PKM2: pyruvate kinase M2
PPARγ: peroxisome proliferator-activated receptor γ
ROS: reactive oxygen species
SMMT: spontaneous mouse mammary tumor
Sp1: specificity protein 1
sRBC: sheep red blood cell
SRC: steroid receptor coactivator
STAT3: signal transducer and activator of transcription 3
TCTP: translationally controlled tumor protein
TfRs: transferrin receptor
TGF-β: transforming growth factor-β
TRAIL: tumor necrosis factor-related apoptosis inducing ligand
uPA: urokinase-type plasminogen activator
VEGF: vascular endothelial growth factor
VM: vasculogenic mimicry
vWF: von willebrand factor
